# Eliminating again, for the last time: A case study of donor support for malaria in Solomon Islands

**DOI:** 10.1002/app5.320

**Published:** 2021-05-04

**Authors:** Camilla Burkot, Katherine Gilbert

**Affiliations:** ^1^ Development Policy Centre, Crawford School of Public Policy The Australian National University Canberra ACT Australia; ^2^ Nossal Institute of Global Health University of Melbourne Melbourne VIC Australia

**Keywords:** disease elimination, foreign aid, Global Fund to Fight AIDS, Tuberculosis and Malaria, malaria, Solomon Islands

## Abstract

Malaria elimination has been a recurring policy goal in Solomon Islands and has historically succeeded in attracting substantial donor support. Drawing on literature review and key informant interviews, we examine the influence of foreign aid on malaria control and elimination efforts in Solomon Islands between 2002 and 2016, as a unique case study of an Asia‐Pacific country with high malaria burden and high donor funding. While aid appears to have contributed to reduced malaria prevalence, the ways in which aid was delivered in the short term had health systems impacts with implications for the elimination agenda. Key areas that will be critical to the future pursuit of malaria elimination in Solomon Islands include: integration of the vertical malaria program, while strengthening provincial‐level service delivery; maximising incentives of performance‐based financing modalities; and policy alignment between donors and domestic actors. We conclude by discussing principles exemplified in the case study of broader relevance to malaria‐endemic countries.

## 1 INTRODUCTION

The elimination of malaria has been a recurring policy objective in Solomon Islands, and a cause which has attracted substantial support from foreign donors. However, the ways in which donor assistance was delivered in the short term have had implications for the viability of the elimination agenda in the long term. As with many other malaria‐endemic countries (Cohen et al., 2012), Solomon Islands has experienced the “boom‐and‐bust” cycle of reductions in incidence followed by periods of resurgence. While the political appetite (both domestic and international) for pursuing malaria elimination in Solomon Islands has proven resilient, achieving elimination in practice has proven highly challenging.

This article seeks to understand the role that donor assistance has played in this boom‐and‐bust cycle, and waxing and waning of the malaria elimination goal, and its impact with a focus on the period 2002 to 2016. It considers: (i) how aid was delivered; (ii) the impact of aid; and (iii) lessons learnt from these efforts, giving greatest attention to this last issue in view of its importance to improving the likelihood of future elimination in Solomon Islands. Understanding the particularities of each malaria‐endemic country's situation will be critical in the lead up to the 2030 regional malaria elimination target.

## 2 METHODOLOGY

A qualitative within‐case methodology was used, involving a review of published and grey literature as well as a series of interviews, which were designed to triangulate findings and fill gaps in the literature. For key periods of time during which investment in malaria increased significantly (particularly 2005 to 2010), there are limited publicly available written records from the Solomon Islands Government and donors, resulting in increased reliance on interview data. This represents both a limitation and strength of the study—it is subject to the bias of the interviewees, but also adds to the information available on the public record.

Interviews took the form of in‐depth, semi‐structured conversations based on a predefined list of topics, which the interviewers (the authors) covered, while probing further in areas most relevant to the interviewee's experience (McIntosh & Morse, 2015). The interview guide covered a range of topics including perceived motivations driving efforts to reduce malaria; the respective contributions and roles played by various aid donors and domestic actors in relation to malaria; the nature of interactions between donors and domestic actors; broader health system changes that occurred as a result of malaria control efforts; and the perceived sustainability of those efforts.

Eighteen interviews were conducted in 2017 with 20 key stakeholders involved in the design, funding, and implementation of malaria control and elimination programs in Solomon Islands. These stakeholders included current and former employees of the Solomon Islands Ministry of Health and Medical Services (MHMS; *N* = 5), bilateral and multilateral donors (*N* = 6), civil society organisations (*N* = 1), and advisers and researchers who worked directly on the malaria program in Solomon Islands in the period of interest (*N* = 8). (In two instances, two interviewees from the same organisation were interviewed jointly.) MHMS interviewees included two officials who had served at the provincial level in the period of interest.

All interview participants provided written informed consent. Some participants also gave consent to audio record the interviews. Where participants did not wish for the interview to be audio recorded, or audio recording was not possible given the location where the interview took place, detailed notes were taken by both authors and combined to produce a single interview record.

All 18 interviews were transcribed and coded for themes by the authors individually, then compared to ensure consistency in themes identified. A framework analysis was used, which involved both coding under the interview guide topics, but also allowing new, unanticipated themes to emerge. Where direct quotes from interviews are presented in this article (shown in italics), they were selected because they were considered either to be illustrative of views expressed by multiple interviewees, or because they offered insights reflective of the interviewee's particular experience in relation to malaria control and elimination in Solomon Islands. As part of the consent process, interviewees selected the level of confidentiality that they desired; most chose full confidentiality, meaning that it is not possible to link the quotations to stakeholder groups.

The findings presented in this article are structured with respect to the three research questions guiding this study (noted above) and are based on a synthesis of the literature review and interview data. While donors and other foreign actors have been engaged in malaria control and elimination efforts in Solomon Islands as far back as the mid‐twentieth century (Burkot & Gilbert, 2017), as noted above this article focuses principally on the period from 2002 to 2016, given the relevance of this period for informing current and future elimination efforts, as well as the availability of data.

## 3 FINDINGS

### 3.1 Delivery of aid

In the early 2000s, Solomon Islands was in a period of flux. Whereas the 1990s had seen a scale‐up of bilateral and multilateral donor interest and a decrease in malaria incidence, an outbreak of ethnic violence in the early 2000s, known as the Tensions, resulted in a macroeconomic crisis and the near collapse of health services, including a major contraction of malaria control efforts (World Bank, 2007).

The entry of the Global Fund to Fight AIDS, Tuberculosis and Malaria (the Global Fund) in 2002 signalled a significant shift in scale. With 10 other Pacific Island countries, Solomon Islands agreed to pursue funding for HIV, tuberculosis (TB) and malaria programs under a regional grant with the Pacific Community (SPC) acting as Principal Recipient (Global Fund, 2002). (While a majority of participating countries had HIV and/or TB and/or malaria, two countries—Solomon Islands and Vanuatu—were classified as malaria‐endemic and agreed to apply for a malaria grant under this arrangement.) Solomon Islands was ultimately successful in receiving contributions from four regional Global Fund grants for malaria control between 2002 and 2015, totalling an estimated US$24.8 million in disbursements, which had a focus on improving access to diagnostics, treatment and long‐lasting insecticidal nets (LLINs) (Global Fund, 2002, 2005, 2007, 2014).

**FIGURE 1:**
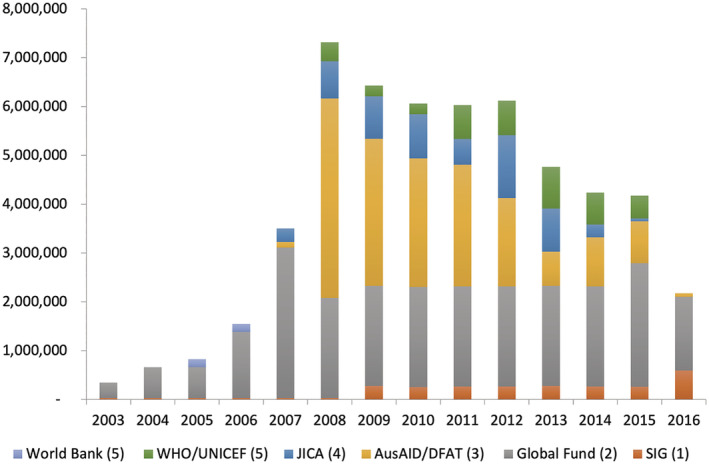
Estimates of Solomon Islands Government and donor contributions to malaria control and elimination, 2003–2016 (US$ current). Sources: (1) Based on NVBDCP budget expenditures (2008–2013) and allocations (2014–2017) from 276 from the recurrent budget of the Solomon Islands Ministry of Finance and Treasury. Estimate (2003–2007) based on trends. Some funds likely for other NBVDCP work areas. Converted to US$ using UN operational exchange rates at December of the given year. (2) Based on Global Fund financial database. Assumes expenditure is consistent throughout grant period. Split between Solomon Islands and Vanuatu based on the relevant grant proposal. (3) Based on OECD CRS (2003–2014). OECD microdata showed that 2014 funds only includes PacMI funds, not Australian Initiative for the Control and Elimination of Malaria (AICEM), which succeeded PacMI in 2014. AICEM expenditure added based on data from the AusTender database and converted to US$ using UN operational exchange rates at December of the given year. (4) Based on OECD CRS. (5) Based on WHO *World Malaria Report 2016* (World Health Organization, 2016)

Influenced in part by the Global Fund, bilateral donors also increased their investment in malaria control and elimination, notably Australia through the Australian Agency for International Development (AusAID). Under the regional Pacific Malaria Initiative (PacMI), launched in 2007, AusAID sought to implement a single consolidated malaria workplan under which the resources and objectives of several donors were to be coordinated, and to drive malaria elimination in specific provinces—in Solomon Islands, these were Temotu and Isabel Provinces (Toole et al., 2010). By 2010, AusAID and the Global Fund resources together comprised approximately 88% of the Solomon Islands National Vector Borne Disease Control Program (NVBDCP) budget (Toole et al., 2010). Figure 1 shows estimated Solomon Islands Government and donor spending on malaria between 2003 and 2016.

At the same time that donor funds dedicated to malaria were growing, there was a parallel interest in wider health system strengthening and reform. A health sector‐wide approach (SWAp) was formed in 2008, intended to support ongoing reforms in health planning and management capacity building, the use of the national health information system, and provincial service delivery (Negin & Martiniuk, 2012). However, its effectiveness was limited by the reticence of both the donor community and the Solomon Islands Government to fully embrace it and transition from their existing ways of working, as reported by Negin and Martiniuk (2012) and echoed by multiple interviewees.

Beginning from around 2014, there were a number of changes in the scale and scope of donor aid for health in Solomon Islands. Policy and budget shifts saw targeted, disease‐specific bilateral aid from Australia significantly wound back in favour of broader investments in core public health systems and capacities (Department of Foreign Affairs and Trade, 2015), which in Solomon Islands were channelled principally through the Health Sector Support Program first established in 2007. The Global Fund revised its allocation method prioritising high‐burden, low‐income countries, leading to reduced funding for Solomon Islands which was classified as a lower‐middle‐income country, discussed further below (Zelman et al., 2016). At the same time, the Solomon Islands MHMS broke from the multi‐country Global Fund grant managed by SPC, and took on the role of Principal Recipient for a single‐country grant beginning in 2015 (Global Fund, 2014; Ruest et al., 2018).

### 3.2 Impacts of aid

**FIGURE 2:**
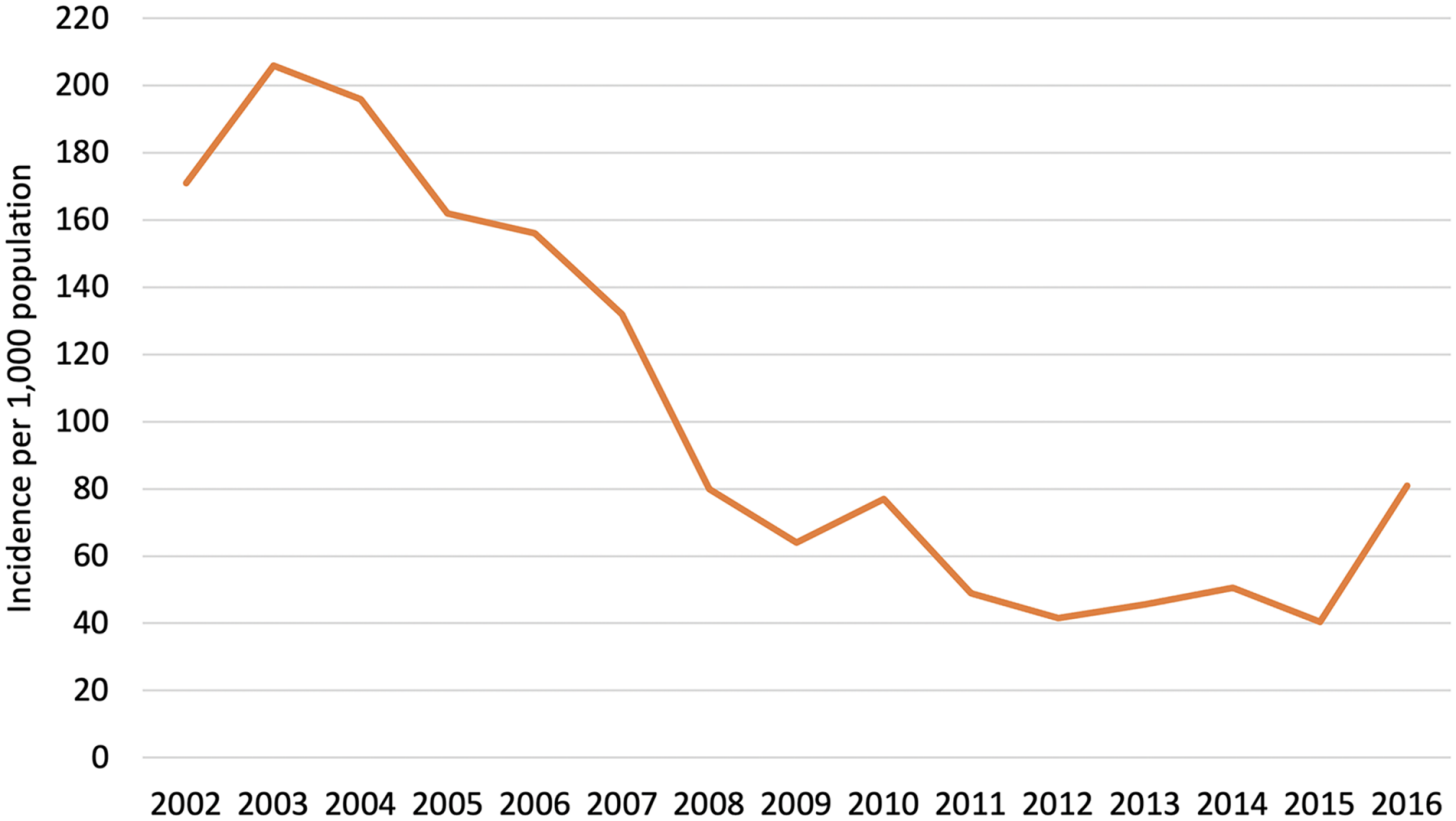
National annual parasite incidence (API), 2002–2016. Source: Solomon Islands Ministry of Health and Medical Services (2017)

In general, respondents and official reports suggest that the donor aid as described above contributed positively to malaria control and elimination efforts. It did so chiefly through enabling the scale‐up of key technical interventions including LLINs and improved case management (using rapid diagnostic tests [RDTs] and artemisinin combination therapies [ACTs]) (MHMS, 2017). Though it is not possible to draw a direct causative relationship between donor aid and disease burden, from 2003 annual national malaria parasite incidence continued on a downward trend, dropping from over 200 to 40 cases per 1,000 by 2015, as shown in Figure 2. As discussed in section 3.2 below, these data were later found to underestimate the national annual parasite incidence (API), but likely still reflect a downward trend.

**FIGURE 3:**
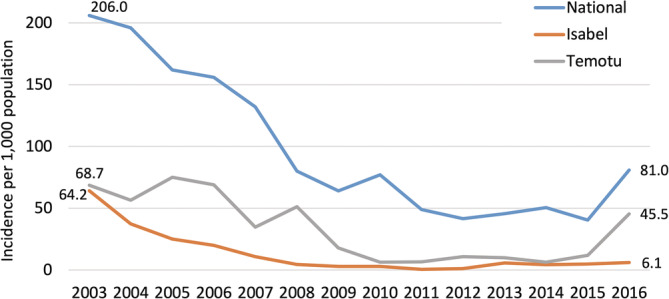
Temotu, Isabel, and Solomon Islands national annual parasite incidence, 2003–2016. Sources: Ministry of Health and Medical Services (2005, 2014, 2017), Office of Development Effectiveness (2009), Over et al. (2004), World Health Organization Western Pacific Region (2011)

Progress towards provincial malaria elimination, about which discussion recommenced in 2007 through PacMI, was uneven and slower than anticipated. Although PacMI initially aimed to eliminate malaria in Temotu and Isabel Provinces by 2014, in that year API was 6.5 per 1,000 persons in Temotu and 4.5 per 1,000 in Isabel (MHMS, 2017). Since then their progress has diverged, as shown in Figure 3.

The ways in which donor aid was delivered triggered a number of long‐term systemic effects described by interviewees. Under the regional Global Fund grants prior to 2015, support was delivered largely in parallel, almost outside of the existing health service delivery systems. The grant was managed at the regional level, and funding flowed to SPC and then directly to the NVBDCP (the program area within the MHMS with responsibility for malaria), rather than the MHMS. This approach reinforced the use of separate systems for service delivery (net distribution, diagnosis and health promotion) and surveillance/information management and resulted in a largely autonomous NVBDCP (commonly referred to as the “Ministry of Malaria”), over which the MHMS Executive exercised little formal control. According to multiple respondents, frustration with this parallel system, combined with the overall reduction in donor funding dedicated to malaria, contributed to the MHMS moving towards greater integration and ownership of malaria programming.

### 3.3 Lessons learnt

#### 3.3.1 Integration of the malaria program and strengthening provincial service delivery

As described briefly above, donor aid granted for malaria in Solomon Islands between 2002 and 2016 was largely vertical and made use of parallel systems. “Vertical” refers to those programs with objectives related to a specific health issue, which often use dedicated systems for governance, finance, and/or service delivery that differ from those used by general health services (Atun et al., 2008; Cairncross et al., 1997; Mills, 2005). Vertical programs often result in the establishment of what are referred to in aid effectiveness literature as “parallel systems” for financial management and delivery of interventions. This was the pattern observed in the delivery of donor support in Solomon Islands, and resulted in what was described by numerous interviewees as a NVBDCP characterised by a high degree of strategic, managerial and financial autonomy from the MHMS.

The regional Global Fund (2003–2014) and Australian (2007–2016) malaria grants were both based on a centralised and vertical model, whereby according to multiple interviewees, subnational level authorities and staff had limited opportunities for strategic influence on the malaria program. The national level parallel systems relied upon to deliver malaria interventions largely circumvented Provincial Health Offices, which resulted in missed opportunities to build the subnational strategic and operational capacity that is now broadly recognised as being essential to malaria control and elimination in the context of diverse and evolving disease burdens.

Interviewees reported that from around 2013 there was a general recognition among MHMS and donor officials that integration of the national malaria program and strengthening of provincial service delivery would be required in order to sustain control and elimination efforts. This shift reflected both external developments (reductions in donor funding globally) and internal reflection (recognition of the limitations of a narrow focus on malaria, versus comprehensive vector‐borne disease control and integrated primary health care). As noted above, the critical importance of Provincial Health Offices to the NVBDCP's continued success in driving down the burden of malaria was also demonstrated by the respective results of elimination efforts in Isabel and Temotu Provinces. Success in Isabel was anecdotally attributed by a number of respondents to strong, broad‐based leadership (encompassing government, church, and traditional leadership structures) and the “*willingness of the [provincial malaria] team*”, although this widely held view has not been empirically interrogated.

As part of broader health service delivery reforms, the MHMS began delegating powers to the provinces, placing greater responsibility for planning, managing, and monitoring service delivery, finances, human resources, assets and information systems at the provincial level. In planning from 2011 but effective from 2016, the MHMS integrated and decentralised its public health programs—that is, disease programs as well as maternal/child health and rural water supply and sanitation (MHMS, 2016a, 2016b). As one of the stronger disease control programs, interviewees described how the NVBDCP was mandated to lead a number of reforms commencing in 2016, including a shift in reporting lines for provincial malaria staff from the NVBDCP to the Provincial Health Director, and shifting responsibility for program implementation (e.g., activities such as LLIN distribution) to the provincial level. Table 1 summarises the changes described by interviewees involved in integrating the NVBDCP back into the broader health system, following MHMS policy change as well as shifts in donor approaches—in particular, the transition from a multi‐country, externally administered Global Fund grant to a national, MHMS‐administered Global Fund grant.

**TABLE 1:** Parallel systems established by the national malaria program (2003–2013) that were integrated into the broader health system from 2016 onwards

WHO health systems building block	Parallel system	Current integration efforts
Governance	Decision‐making through regional, donor‐funded bodies	Decision‐making through MHMS Executive Board and national Global Fund Country Coordinating Mechanism
	One malaria plan, but separate (donor‐aligned) planning and budgeting cycle	One malaria plan, integrated into national and provincial planning and budgeting cycle
	Program‐specific supervision visits to facilities	Integrated supervision by Provincial Health Office
	Program‐specific assets (boats, outboard motors, vehicles)	Integrated management of assets by Provincial Health Office
Financing	Funds disbursed to development partner account but managed by the NVBDCP or a private trustee	Funds flow to development partner account and managed in accordance with Solomon Islands government systems, but with additional controls on spending due to 2013 fraud
Service delivery	Diagnostic testing administered by community‐based microscopists financed by the program	Diagnostic testing administered by nurses financed by health system
	Program‐specific community based health promotion	Integrated Healthy Village program led by MHMS health promotion unit
Human resources	Donor‐funded staff in malaria program management (approx. 7) and service delivery (approx. 100), specifically for community‐based microscopy	Some program management positions have been incorporated into the MHMS payroll within the human resource and finance divisions. The community‐based microscopists program ended (i.e., none transitioned to MHMS payroll)
	Program‐specific international technical assistance (TA) contract	TA reduced and integrated into broader MHMS/DFAT TA contract
Supply	Procurement by Global Fund	Continued but program now ordering directly through online Global Fund platform wambo.org
	Diagnostics and treatments distributed through national supply chain (National Medical Stores to second level medical stores, and Provincial Health Office to facility), but with informal program support	Malaria program no longer supports distribution from second level medical stores to facilities but is now collecting stock levels from facilities
	Bed nets shipped by Global Fund directly to Provincial Health Office, then program distributes to communities	Continued, but Provincial Health Office now responsible for distribution
Information	Data specified by program, collected by microscopist or community‐based microscopists at facility and entered into Malaria Information System by malaria information officers at Provincial Health Office	Data specified by program (using malaria case management register), collected by nurses at facility and entered into DHIS2 by malaria information officers at Provincial Health Office

This reform process revealed weaknesses in both national and provincial systems, and mixed views among interviewees on the impact and appropriate role of donors. At the national level, there was recognition among MHMS and donor officials that the decade‐long investment in health systems by the Australian aid program through the SWAp significantly improved the health information and supply systems, making integration smoother than it otherwise would have been. At the provincial level, there was broad appreciation that stronger subnational capacity to plan and implement interventions will be critical to the success of future malaria control and elimination efforts, particularly in light of significant variation in malaria burden between provinces.

#### 3.3.2 Incentives and performance‐based financing

Performance‐based financing mechanisms, perceived as a way to strengthen effectiveness and accountability, have become increasingly common in global health aid. With a reduced Global Fund allocation for Solomon Islands, and a MHMS that had been attempting to break from the regional grant and become Principal Recipient of its grant for some time, an opportunity arose in 2015 to restructure the grant. In 2015 the Global Fund and Solomon Islands signed a national malaria grant with MHMS as Principal Recipient, using a hybrid Cash on Delivery funding model. Most of the MHMS officials and donor partners interviewed welcomed this development, perceiving it as an opportunity to strengthen country ownership and increase use of MHMS systems.

The Global Fund Cash on Delivery model as implemented in Solomon Islands included a traditional grant and a performance‐based financing component: while a significant proportion of the grant is expended directly on LLINs, drugs and diagnostic tests through the Global Fund's Pooled Procurement Facility, the remaining funds are disbursed retroactively by the Global Fund only if predetermined performance targets are met (Global Fund, 2014). The grant was also subject to the Global Fund's then “willingness to pay” policy, in which 15% of the Global Fund grant allocation was contingent on Solomon Islands demonstrating increasing co‐financing of the program.

The performance of the Cash on Delivery grant was assessed using indicators that are specified in the agreement between the Global Fund and the MHMS. For the first year of the Global Fund grant commencing in 2015, the target was based on reporting completeness; in subsequent years, national API was the target that determined reimbursement eligibility (Global Fund, 2015). After API stabilised at 40 per 1,000 in 2015, it doubled to 81 per 1,000 in 2016 (MHMS, 2017). A subsequent independent review conducted on behalf of the Global Fund concluded that this increase was likely due to a combination of factors including improved diagnostics and surveillance, as well as an increase in real transmission (Ruest et al., 2018). Thus, the use of more sensitive tools and efforts to improve data accuracy had the contradictory effect of potentially jeopardising reimbursement prospects under the Cash on Delivery model.

The Cash on Delivery model requires the Principal Recipient (in this case, the Solomon Islands Government as represented through MHMS) to “frontload” (pre‐finance) the performance‐based component of the grant, increasing the government's incentives to reach the targets and receive reimbursement. To some extent, incentivising performance rests on the MHMS bearing the responsibility for frontloading the performance component of the grant, so that it carries the risk of non‐reimbursement if the targets are not met. However, this has not necessarily been the case in practice: according to interviewees, in 2015 unspent Australian funds were used to frontload the grant on behalf of the Solomon Islands Government, and in 2016 and 2017 the surplus of unspent Global Fund funds were used.

The issue of risks and incentives was also raised by interviewees in relation to the management and transfer of funds between national and provincial levels. Whereas previously provincial malaria personnel were dependent on grants from the NVBDCP to implement specific, agreed activities, in 2017 the NVBDCP commenced a new approach of allocating block grants directly from the national program to provinces. Ostensibly this was intended to enable provincial malaria personnel greater financial control and flexibility over implementation. However, according to interviewees, uncertainty around the process of raising and disbursing these funds, and delays in the Ministry of Finance, led to slow disbursement. Provincial Health Offices provided stopgap financing for malaria activities in the midst of these delays, but had reservations, according to one interviewee, about whether performance‐based reimbursement from the Global Fund would eventuate.

#### 3.3.3 Donor/recipient alignment

A third area for consideration is the extent to which donors and the Solomon Islands Government have been aligned in their understanding of the implications of adopting malaria elimination as a policy goal, and the strategy for achieving it. The interviews conducted for this study broadly suggest that the pursuit of elimination in Solomon Islands followed a renewed global push for elimination, which began to gain traction in the mid‐2000s. On the donor side, the global dialogue around elimination—connected principally to the development of more effective insecticide‐treated nets, diagnostics and drugs—gave renewed credence to the idea that malaria elimination was possible, a view which subsequently filtered down to the Pacific and Solomon Islands. The decision to establish PacMI and increase the quantum of funding dedicated to malaria in the Pacific region can be traced to the 2006 White Paper on the Australian aid program, framing malaria as a high‐level policy priority of the Australian Government (AusAID, 2006). A global technical adviser interviewee connected the establishment of PacMI to AusAID's desire at the time to fund “*innovative, boutique*” initiatives, which would both have an impact in the Pacific and position Australia as a global development leader. Similarly, in 2005 the Global Fund encouraged countries to increase the size of their funding applications, reflecting pressure it was under from its donors to spend. Despite hand‐wringing among those for whom the failed 1960s Global Malaria Eradication Program still loomed large—as one researcher recalled, at the time “*there was a lot of anxiety about whether [elimination] is something we should all be embracing, and whether we were setting ourselves up to fail*”—donor interest in rekindling elimination picked up momentum.

This donor interest in elimination translated effectively into interest and professed commitment at the political level in Solomon Islands, with the prime minister and other high‐level government representatives making public statements of commitment to the regional and global malaria elimination goals. However, while most interviewees for this study (who have been engaged in malaria in Solomon Islands at the operational level) attested that the goal of elimination is “*strong in the minds of the [national malaria] program and the Ministry [of Health]*” and that the government is politically obliged to “*go along*” with regional and global malaria elimination initiatives, nearly all agreed that a 2030 deadline for achieving malaria elimination in Solomon Islands is unrealistic. 1

In reflecting on political statements of support for elimination in Solomon Islands, several interviewees identified an apparent perception gap between political aspirations and understanding of implementation capacity: “*[Solomon Islands Government] signed for it, but they don't understand it—what is required in order to actually carry out or enter into elimination*.” However, some suggested that donors have been equally underinformed about the realities of pursuing elimination in Solomon Islands, perceiving that they have tended to not adequately grasp the specifics of this context as compared to other malaria‐endemic countries. For example, discussing challenges with LLIN distribution, one interviewee concluded: “*[Donors] think that this place is one island. They always compare us with Africa. But Africa is a totally different ball game*.”

These gaps in understanding were not entirely unknown to individuals working on malaria elimination at the time, and some steps were taken to seek to improve donor/recipient dialogue. Though not limited to malaria, the formation of a SWAp reflected recognition by donors of the need for regular dialogue, in line with global development effectiveness principles. However, interviewees identified practical challenges in ensuring smooth implementation of the SWAp, with higher‐level structural conditions (e.g., poor alignment of planning and budgeting cycles between donors and the Solomon Islands Government) representing a major barrier to coordination. Specific to malaria, a “Malaria Reference Group” was formed under PacMI with representation from the donor (AusAID), MHMS and technical experts—a structure which should have served to enable pragmatic dialogue and coordination at both the strategic and operational levels. However, some interviewees reflected that in practice the power dynamics in this group prevented these discussions from fully grappling with the operational constraints faced by the NVBDCP and MHMS.

Alignment on policy and high‐level objectives is one thing, but strategy is another. To be feasible, an elimination strategy must encompass not only epidemiological, health systems and financial realities, but also broader health ministry priorities and community perceptions of malaria. On the latter point, several interviewees remarked that, in their experience, malaria is no longer commonly perceived by Solomon Islanders as a serious health threat. The extent to which community perceptions of the urgency of malaria elimination (or lack thereof), as compared to other health priorities, have historically been taken into consideration by donor and government partners when designing malaria programming is unclear.

## 4 DISCUSSION

### 4.1 The Global Fund, health systems strengthening, and transition

Mirroring the trend observed in Solomon Islands, donor aid for malaria has been in decline globally since 2010, and the decreases in donor funds have not been replenished by increases in government health expenditure (Shretta et al., 2017). This reinforces the need to ensure the most effective use of available resources, and highlights the risk that gains in progress towards elimination may be lost if donor assistance is phased out prematurely. Yet while global aid for malaria has declined, the Global Fund recently held a successful replenishment, achieving $14 billion in donor pledges in October 2019—the largest in its history. The Global Fund is now the largest donor for malaria, providing 65% of all international (and 42% of total) malaria financing (Global Fund, 2019c). This places increasing importance on the Global Fund's approach to achieving malaria elimination.

An inherent tension exists in the Global Fund's approach, highlighted in this article, between its specific disease focus and its growing commitment to health systems strengthening (HSS). Although the Global Fund's founding principles set out in its Framework Document included a commitment to supporting programs that “address [HIV, tuberculosis and malaria] in ways that will contribute to strengthening health systems” (Global Fund, 2001, p. 93), the extent to which the Global Fund has achieved this in practice has been questioned (Sherry et al., 2009; World Health Organization, 2007). The Global Fund has also shifted and modified its approach to HSS since its inception: from standalone HSS grants to integrated HSS grant components under the banner of “resilient and sustainable systems for health” (RSSH); and from an emphasis on a “diagonal approach” (a middle path in the vertical versus horizontal programming debate) to “do no harm” and “equity” (Global Fund, 2019a).

Common themes emerge in reviews of the Global Fund's HSS efforts. A report by the Global Fund's own Technical Review Panel found that 75% of Global Fund RSSH funding requests submitted in the 2017–19 grant cycle focused more on health systems support and program and grant management costs (Technical Review Panel, 2018). That is, investments have been concentrated more on health system inputs, such as supplementing human resources (predominantly health worker training and salary support), supplies and information systems, rather than systems strengthening and sustainability functions, such as improving governance, policy development and financial management capacity (Fan et al., 2017; Technical Review Panel, 2018; Warren et al., 2013). Moreover, a review of Global Fund HSS spending to 2014 found that while investment in HIV and tuberculosis was associated with Global Fund HSS investment, this was not the case for malaria grants (Fan et al., 2017). This raises the question whether there is a difference in the nature or history of malaria versus HIV and tuberculosis programming that rendered malaria programs less likely to pursue HSS investment.

The current *Global Fund Strategy 2017–2022: Investing to End Epidemics* positions “building resilient and sustainable systems for health” as a core strategic objective (Global Fund, n.d.), and is accompanied by an information note to inform RSSH applications for the next funding cycle (Global Fund, 2019a). The information note and associated modular framework are broad in scope and list a variety of RSSH activities across different health system components, similar to the World Health Organization's “building block” approach (Global Fund, 2019a). The challenge for applicants and the Global Fund is to ensure that RSSH grants (or grant components) are based on robust health systems analyses, with activities linked to a larger evidence‐informed and context‐driven strategy that addresses cross‐cutting constraints.

There is also potential contradiction between HSS approaches and the Global Fund's emphasis on results and, in particular, its Cash on Delivery model. HSS approaches need time for learning by doing, to draw on what's working and adapt in response to unintended consequences—which may require longer timeframes than performance‐based financing models allow. Knowing this, applicants may be less inclined to undertake complex HSS as opposed to simpler health systems support. There is thus room for the Global Fund to consider how to incentivise applicants to favour HSS approaches. Under a Cash on Delivery model, inclusion of a further performance‐based indicator relating to program execution may help to incentivise higher levels of expenditure (and, by extension, any systems reforms required in order to facilitate this expenditure).

The impact of Global Fund's policies on malaria elimination in Solomon Islands of course will depend on the extent to which it continues to be a significant donor there. Under the Global Fund's transition policy, upper‐middle‐income countries (UMIC) are only eligible for grants if the country has a high disease burden. However, its “small islands economy exception”, a mirror of the World Bank's loan exception policy, grants eligibility to Pacific Island countries that are classed as UMIC regardless of their disease burden (Global Fund, 2019b). The exception applies to Solomon Islands but not Papua New Guinea (World Bank, 2019). Thus, even if Solomon Islands reaches UMIC status over the next 10 years, it will remain eligible for malaria grants from the Global Fund for the foreseeable future.

This raises the question of whether the Global Fund is prepared to remain the major financier of malaria elimination in Solomon Islands, and if so the manner in which it will invest. While the Global Fund's investment in Solomon Islands has transformed over the past five years (as described above), it remains heavily centralised; documents for the current grant note that the Global Fund is working at the national level, while the NVBDCP at provincial level. Although Australian bilateral health sector support is being increasingly directed to the provincial level, it is unclear if this “division of labour” will be sufficient to meet the recognised need to build capacity at the provincial level in order to achieve elimination.

### 4.2 Prioritisation

Economic modelling demonstrates the expected positive, long‐term economic benefits of malaria elimination (e.g., Shretta et al., 2019). However, the achievement of malaria elimination is a long‐term goal—with distant, hypothetical benefits, and a process that is likely to require several political cycles—that must be weighed against short‐term priorities, that is health issues that are more visible and/or prevalent. Allocation of resources to health priorities that have differing long‐ and short‐term benefits is a challenge faced to some degree by all governments, but one which is particularly acute for low‐ and middle‐income countries.

The extent to which the availability of donor funds influences the prioritisation of health issues, and correspondingly resource allocation, by recipient country governments has been studied with particular reference to the global health initiatives (GHIs), such as the Global Fund and Gavi, the Vaccine Alliance, given the scale of their investment. However, the implications are likely to translate to targeted, disease‐specific funds from other types of donors (bilateral, multilateral development banks, etc.). Although the establishment and growth of GHIs significantly increased awareness of and resource availability for specific diseases, the extent to which they have influenced recipient countries' health priorities has been the subject of critique (The Lancet, 2009). A 2009 review of the interactions between GHIs and country health systems concluded that while GHIs contributed to an aggregate increase in overall health financing, the allocation of GHI disease‐specific funding was not always aligned with countries' stated priorities, health sector strategies, or burden of disease (Samb et al., 2009).

GHIs have generally increased the resource envelope for health in countries including Solomon Islands, but in a way that potentially risks the distortion of funding allocations. That is, they increased the resource envelope but only for specific disease areas (e.g., malaria), rather than increasing the whole health sector envelope in the way that sector budget support would do. On the one hand, this may serve recipient countries by allowing them to redirect their existing domestic resources to other disease areas that are not identified as donor priorities. However, the attachment of specific co‐financing requirements—intended to strengthen accountability and sustainability—may prevent countries from fully maximising the use of available resources by tying portions of their existing resource envelope to the donor funds. In effect, this may further stretch already limited health budgets by reducing the availability of domestic funds for priorities other than those selected by the GHI. This is problematic as while donors are entitled to seek accountability for the funds disbursed, equally they have a responsibility to identify mechanisms for disbursement that minimise distortion and do not impose unnecessarily burdensome requirements (i.e., co‐financing, administrative, reporting) on the recipient.

In settings where medium‐ to long‐term economic growth is anticipated and/or where health makes up a relatively low proportion of total domestic government expenditure, co‐financing represents a rational policy to promote sustainability and eventually a full transition from donor financing. Unfortunately, this is not the case in Solomon Islands, which like most other Pacific Island countries faces modest economic growth prospects, coupled with high population growth projections (Anderson et al., 2014). The most up‐to‐date World Development Indicators data (2018) show that domestic public health sector expenditure in Solomon Islands represents 7.9% of total general government expenditure (World Bank, 2021). This is on par with other Pacific Island small states (8.1%), but higher than aggregated lower‐middle‐income countries (4.9%) (World Bank, 2021).

Further, although the proportion of total health expenditure in Solomon Islands accounted for by external sources has declined from a high of over 40% in 2011, by 2016 external resources continued to represent over a quarter (26.2%) of total health expenditure (Ruest et al., 2018). While domestic financing for malaria has increased (including the first Solomon Islands Government budget contribution to program operations in 2016) and there remains a formal political commitment to the goal of malaria elimination in Solomon Islands, donor funding is expected to remain a significant source of health finance for the foreseeable future (Ruest et al., 2018). In such a context, where donor financing figures so prominently in overall health expenditure (despite talk of transition), the need to align donor and recipient country health priorities is not a challenge that is likely to dissipate in the short to medium term.

### 4.3 Regional mechanisms

Another feature of the history of donor support for malaria elimination in Solomon Islands has been the use of regional mechanisms. As described above, these have come in the form of dedicated funding administered through a regional organisation (e.g., Global Fund multi‐country grant managed by SPC), and capacity‐building projects structured at a regional level (e.g., Australian bilateral funding for malaria and other vector‐borne diseases).

Since its inception, the Global Fund has accepted regional/multi‐country grant proposals. 2 In a review of multi‐country grant proposals in Rounds 1 to 10, Smith Gueye et al. (2012) report that successful proposals presented a strong argument for the use of a multi‐country approach, highlighting the relevance of international cross‐border collaboration as well as a “‘network approach’ by which benefits are derived from economies of scale or from enhanced opportunities for mutual support and learning or the development of common policies and approaches” (Smith Gueye et al., 2012). However, they also found that multi‐country proposals have historically been less successful than single‐country grants in securing Global Fund support: of 16 multi‐country malaria grants submitted between Rounds 1 and 10, only six were funded—three of which were grants to Solomon Islands and Vanuatu.

Consideration of regional mechanisms is particularly pertinent in the Pacific, where the concept of regionalism has a long history (Bryar & Naupa, 2017). While Pacific regionalism has primarily been debated in the context of Pacific countries' prosecution of their shared political and economic interests on the global stage, the effectiveness of regional approaches to deliver sustainable development outcomes within the region is contested. A 2014 review of Pacific regional service delivery initiatives (including several health sector initiatives) concluded that these tend to underperform from a development effectiveness perspective (Dornan & Newton Cain, 2014). This is attributed to the challenges inherent in sustaining voluntary cooperation as well as the diversity and capacity constraints facing Pacific Island countries.

These factors—diversity and capacity constraints—pose additional challenges in the context of malaria elimination, as these exist not just at the national level but extend to the subnational level. The Global Fund Pacific multi‐country grants for malaria, in which Solomon Islands participated between 2003 and 2014, could be described as a service delivery quasi‐regional initiative—it represented a regional approach to grant administration (managed through a regional institution, SPC), which benefited from globally pooled procurement of commodities, but service delivery remained the responsibility of the individual countries incorporated under the grant. Similarly, the Australian‐funded PacMI program was framed using a regional lens, and although it succeeded in periodically bringing together representatives from different countries to share experience, its primary focus was capacity building at the national level.

As such, it is unclear whether either example succeeded in capitalising on the “network” approach described above as a primary objective and defining feature of regional approaches—but equally unclear whether such an approach would offer significant benefits above nationally focused approaches, particularly at this stage of Solomon Islands' journey towards malaria elimination. This is consistent with the findings of Zelman et al. (2016), who reviewed the impacts of the Global Fund's New Funding Model for financing allocations to malaria‐eliminating countries, including impacts on regional grants. They noted that while regional grants may address gaps in specific technical areas that are not readily addressed by national programs (e.g., cross‐border surveillance), they are insufficient to achieve malaria elimination: “Without strong national malaria programmes, [Global Fund] regional grants may be less effective in achieving regional goals” (Zelman et al., 2016, p. 15).

While it is possible that regional grants may play a supportive role in moving countries closer to elimination—as evidenced by the progress made in the Greater Mekong Subregion over the life of the Global Fund's Regional Artemisinin‐resistance Initiative, for example—evidence for their effectiveness remains limited (Lover et al., 2017). Any benefits that might be offered by a regional approach (e.g., in relation to reduced procurement or transaction costs) need to be weighed against the importance of enhancing country ownership and alignment to national health service delivery and financing systems. Similarly, in being organised around a single disease, regional funding mechanisms have tended to reinforce a vertical disease focus. For Solomon Islands, this is contrary to MHMS efforts to promote greater integration and the delivery of comprehensive primary health care. Thus, it is less likely that regional financing or service delivery mechanisms will be palatable unless donors can demonstrate how such mechanisms can integrate into and complement national systems.

## 5 CONCLUSION

This case study of donor aid for malaria in Solomon Islands has shown that while significant progress was made between 2002 and 2016, substantial work remains to achieve malaria elimination. It has also shown that donors—principally the Global Fund but also other bilateral and multilateral partners—have exercised considerable influence over the policy goals and strategic direction towards malaria elimination, which affected the broader health system in Solomon Islands. As is often the case in aid and development policy, these effects have been neither wholly positive nor negative, and have occasionally produced unexpected consequences. Key lessons learnt, drawn from a review of literature and the views of stakeholders with direct experience of implementation in Solomon Islands, suggest that efforts underway to integrate the national malaria program and strengthen provincial service delivery should be continued and actively supported by donors, who should also seek to better align malaria elimination goals, strategies and financing to the priorities of the MHMS and Solomon Islands Government. Doing so will help ensure that the next push to achieve malaria elimination in Solomon Islands will be the last. Failure to do so would almost certainly result in another resurgence of malaria—entailing further preventable morbidity and mortality, the loss of the substantial investments made in driving down malaria to date, and potentially the development of resistance of the malaria parasite and vector to existing effective drugs and insecticides—in a global context that offers little certainty of continued political and financial commitment to the cause of malaria elimination.

## Data Availability

The interview data that support the findings of this study are available on reasonable request from the corresponding author. The data are not publicly available due to privacy and ethical restrictions.

## Footnotes

^1^
To this end, it is worth noting that the current National Malaria Elimination Roadmap (copy on file with authors) adopted in 2019 aims for zero transmission of malaria in Solomon Islands by 2030, with World Health Organization (WHO) certification of elimination status by 2033.

^2^
Note: Global Fund terminology shifted from a distinction between “multi‐country” and “regional” grants under the 2014–16 funding round, to simply referring to “multi‐country” grants in the 2017–19 funding round (Global Fund, 2017).
